# More Favorable Metabolic Impact of Three-Times-Weekly versus Daily Growth Hormone Treatment in Naïve GH-Deficient Children

**DOI:** 10.1155/2017/8469680

**Published:** 2017-05-28

**Authors:** Alessandro Ciresi, Floriana Cicciò, Stefano Radellini, Valentina Guarnotta, Anna Maria Calcaterra, Carla Giordano

**Affiliations:** Section of Endocrinology, Biomedical Department of Internal and Specialist Medicine (DIBIMIS), University of Palermo, Palermo, Italy

## Abstract

**Objective:**

To evaluate whether two different regimens of weekly injections could lead to similar auxological and metabolic effects in children with growth hormone deficiency (GHD).

**Design:**

32 GHD children (25 males, mean age 10.5 ± 2.2 yr) were randomly assigned to receive daily (group A, 16 patients) or TIW (group B, 16 patients) GHT for 12 months.

**Methods:**

Auxological parameters, insulin-like growth factor-I (IGF-I), glucose and insulin during OGTT, glycosylated hemoglobin (HbA1c), lipid profile, the oral disposition index (DIo), the homeostasis model assessment estimate of insulin resistance (Homa-IR), and the insulin sensitivity index (ISI).

**Results:**

After 12 months, both groups showed a significant and comparable improvement in height (*p* < 0.001) and IGF-I (*p* < 0.001). As regards the metabolic parameters, in both groups, we found a significant increase in fasting insulin (*p* < 0.001 and *p* = 0.026) and Homa-IR (*p* < 0.001 and *p* = 0.019). A significant increase in fasting glucose (*p* = 0.001) and a decrease in ISI (*p* < 0.001) and DIo (*p* = 0.002) were only found in group A.

**Conclusions:**

The TIW regimen is effective and comparable with the daily regimen in improving auxological parameters and has a more favorable metabolic impact in GHD children. This trial is registered with ClinicalTrials.gov NCT03033121.

## 1. Introduction

Children with acclaimed GH deficiency (GHD) should be treated with recombinant human GH with the primary objective of the normalization of height and attainment of normal adult stature. In addition, the metabolic and body composition benefits should always be considered. Long-term GHT should theoretically mimic the complex physiological pattern of GH release, which is unlikely to be achieved with the current modes of administration. From 1960, GH treatment (GHT) in hypopituitary children was restricted for many years to three injections per week (TIW), because it was convenient and efficacious [1]. The availability of recombinant GH has enabled larger scale use of GHT on a daily basis. Indeed, daily subcutaneous injections were found to be more effective for children and this daily format was introduced as a routine treatment regimen during the 1980s [2]. Currently, GHT in GHD patients is commonly administered in daily injections [3], but this modality of treatment is not complied with by the totality of patients. In addition, it should be considered that physiological spontaneous pulsatile GH secretion cannot be reproduced by either TIW or daily injections and that a perfect physiological regimen has not been identified. The role played by GH pulsatility on both growth and metabolism is important, although it is not known whether closer imitation of the endogenous GH secretory pattern would improve the response to GHT [4]. For these reasons, efforts have been made to make GHT more similar to physiological GH secretion and long-acting forms of recombinant human GH with a more convenient dosing regimen are under development for children and adults [5–7].

In addition, the effectiveness of any therapy is contingent on patient adherence and the reported levels of nonadherence to GHT in children are highly variable, leading to reduced efficacy and increased health care costs [8–11]. Since one of the main factors associated with poor adherence to GHT is the complex treatment regimen, reduced frequency of the administration of medication is generally associated with better adherence [12]. The clinical outcomes of daily versus TIW GH administration were already studied many years ago. The TIW regimen seems to produce similar effects to those of daily injections in both adults and children with GHD [13–15], although several studies have reported that the daily GH administration in children was more effective than TIW dosing on linear growth [16, 17]. However, to date, the metabolic effects of GHT given in an alternative regimen in GHD patients have only been evaluated in adults and never in children. This study aimed to reevaluate whether the same weekly dose of GH given by two different regimens of weekly injections could lead to similar auxological and especially metabolic effects in children with GHD.

## 2. Subjects and Methods

Thirty-two prepubertal children (25 males, 7 females; mean age 9.5 ± 2.2 years) with a diagnosis of isolated idiopathic GHD were consecutively enrolled in this prospective randomized clinical study over a period going from January 2015 to December 2015 from the Section of Endocrinology, University of Palermo (www.ClinicalTrials.gov, identification number NCT03033121).

All subjects and their parents gave informed written consent to the study. The diagnosis of GHD was established by the clinical, auxological, radiological, and biochemical criteria of the GH Research Society [3].

As auxological data, we considered the height and growth velocity 1 year before diagnosis. As radiological criteria, we considered a bone age delay, estimated from an X-ray of the left wrist and hand and evaluated according to the methods of Greulich and Pyle, of at least 1 year with respect to the chronological age [18]. Subsequently, we calculated the bone/chronological age ratio.

Biochemically, GHD was demonstrated by the failure of GH to respond to the two stimuli (arginine and glucagon test) with GH peaks below 8 *μ*g/L. We excluded children affected by multiple pituitary hormone deficiency or receiving any other kind of hormonal replacement therapy or drug and GHD children treated for less than 12 months. All children, including older ones, were in the first stage of sexual development according to the Marshall and Tanner criteria [19] to avoid any interference of puberty on auxological and metabolic parameters, and they maintained the prepubertal hormonal status during the 12 months of follow-up. Neuroimaging, with magnetic resonance imaging of the hypothalamic-pituitary region, was arbitrarily performed in GHD children with more severe GHD, that is, with GH peak ≤3 *μ*g/L (13 children). No evidence of intrasellar lesions was found, while 5 children showed pituitary hypoplasia.

Enrolled patients were consecutively and randomly assigned by using the balance block randomization method in a 1 : 1 ratio to two different GHT regimen, given in the evening: group A included 16 children (12 males, 4 females, mean age 9.8 ± 2.2 years) receiving daily GH injections, and group B included 16 children (13 males, 3 females, mean age 9.3 ± 2.2 years) receiving TIW of GH.

All children were naïve to GHT. In line with our internal protocol, in all children, we used a weight-based GH treatment [20]. Regardless of GH peak, we used the same initial weekly dose of 0.175 mg/kg (corresponding to the daily dose of 0.025 mg/kg) of GH with a gradual increase every 6 months in order to maintain the IGF-I levels constantly in the normal range. In detail, from months 1 to 6, we used the mean weekly dose of 0.175 mg/kg and from months 6 to 12, the mean weekly dose of 0.20 mg/kg.

Four children (3 males, 1 female; 2 from group A and 2 from group B, resp.) who changed pubertal stage (from the first to the second stage) during the follow-up were excluded from the study.

### 2.1. Study Design

In all patients, at baseline and after 12 months of GHT, we measured the body height (standard deviation, SD), body mass index (BMI), and waist circumference (WC). The height and BMI were expressed as SD due to the wide age range of patients.

Blood samples were drawn after an overnight fast. Laboratory assessment included fasting glucose and insulin levels, insulin-like growth factor-I (IGF-I), glycosylated hemoglobin (HbA1c), lipid profile including total cholesterol, high-density cholesterol (HDL), and triglycerides. Low-density lipoprotein (LDL) cholesterol levels were evaluated by the following formula: total cholesterol − (HDL cholesterol − triglycerides/5). This sample also served as the baseline sample for an oral glucose tolerance test (OGTT). Blood samples were collected every 30 min for 2 h for glucose and insulin measurements. At 12 months, in group B, all samples were drawn the morning after the GH dose was received.

The estimation of the basal insulin secretion included fasting insulin levels, while the oral disposition index (DIo) was used as an index of the ability of the *β*-cell to regulate its insulin response to stimuli based on the differences in insulin sensitivity. DIo was calculated at the times 0′ and 30′ during OGTT as described [21]. As surrogate estimates of insulin sensitivity, we considered the homeostasis model assessment estimate of insulin resistance (Homa-IR) [22] and the insulin sensitivity index (ISI), a composite index derived from the OGTT and validated by Matsuda and DeFronzo [23].

The institutional Ethics Committee of the University of Palermo approved this study. At the time of hospitalization, a written informed consent for the scientific use of the data was obtained from all the participants' parents.

### 2.2. Hormone and Biochemical Assays

All biochemical data were collected after an overnight fasting. Glucose, HbA1c, and lipids were measured in the centralized accredited laboratories of the University of Palermo with the standard methods. Serum insulin was measured by ELISA (DRG Instruments GmbH, Germany). The sensitivity of the method was 1 IU/mL. The normal insulin range (IU/mL) was 5–19. Throughout the follow-up, serum GH levels were measured by immunoradiometric assay using commercially available kits (Radim, Italy). The sensitivity of the assay was 0.04 *μ*g/L. The intra- and interassay coefficients of variation (CVs) were 2.5–3.9 and 3.8–5.0%, respectively. We reported GH concentrations in *μ*g/L of IS 98/574.

IGF-I levels were measured by a chemiluminescent immunometric assay (Immulite 2000; Diagnostic Products Corp., Los Angeles, CA) using murine monoclonal anti-IGF-I antibodies. The standards were calibrated against the World Health Organization second IS 87/518. The assay had an analytical sensitivity of 1.9 *μ*g/L. The intra- and interassay CVs were 2.3–3.9% and 3.7-8.1%, respectively.

### 2.3. Statistical Analysis

The Statistical Packages for Social Sciences (SPSS) version 17 was used for data analysis. Baseline characteristics were presented as mean ± standard deviation (SD); rates and proportions were calculated for categorical data. The normality of distribution of the quantitative variables was assessed with the Kolmogorov-Smirnov test. The differences between groups were evaluated by the Student *t*-test. A *p* value of <0.05 was considered statistically significant.

## 3. Results

The baseline clinical and biochemical features of children, grouped according to the different GHT regimens, are shown in Table 1.

No difference was found for chronological age (9.8 ± 2.2 versus 9.3 ± 2.2 years; *p* = 0.553), height (−2.06 ± 0.43 versus −2.04 ± 0.69 SD; *p* = 0.332), growth velocity (−3.4 ± 0.9 versus −3.3 ± 0.9 SD; *p* = 0.418), BMI (−0.46 ± 0.48 versus −0.79 ± 0.45 SD; *p* = 0.418), WC (60.5 ± 10.4 versus 60.1 ± 9.0 cm; *p* = 0.828), and bone age delay (0.81 ± 0.12 versus 0.81 ± 0.09; *p* = 0.053) between groups A and B. Similarly, the 2 groups of children showed similar IGF-I levels (99.5 ± 23.8 versus 100 ± 23.4 *μ*g/L) and GH peak after arginine (5.08 ± 2.54 versus 3.28 ± 2.49 *μ*g/L; *p* = 0.062) and glucagon test (3.22 *μ*g/L ± 1.96 *μ*g/L versus 4.77 *μ*g/L ± 3.31 *μ*g/L; *p* = 0.119).

After 12 months of treatment, both groups of children showed a significant increase in height (−1.5 ± 0.40 versus −2.06 ± 0.43 SD and −1.5 ± 0.5 versus −2.04 ± 0.69 SD; both *p* < 0.001), growth velocity (2.7 ± 0.7 versus −3.4 ± 0.9 SD; *p* = 0.003 and 3 ± 1.4 versus −3.3 ± 0.9 SD; *p* < 0.001), and IGF-I levels (332 ± 125 *versus* 99.5 ± 23.8 *μ*g/L and 352 ± 89.1 versus 100 ± 23.4 *μ*g/L; both *p* < 0.001), with a concomitant lower, although not statistically significant, bone age delay (0.90 ± 0.05 versus 0.81 ± 0.12; *p* = 0.089 and 0.89 ± 0.11 versus 0.81 ± 0.09; *p* = 0.098), while no significant difference was found in BMI and WC (Table 1).

**Figure 1:**
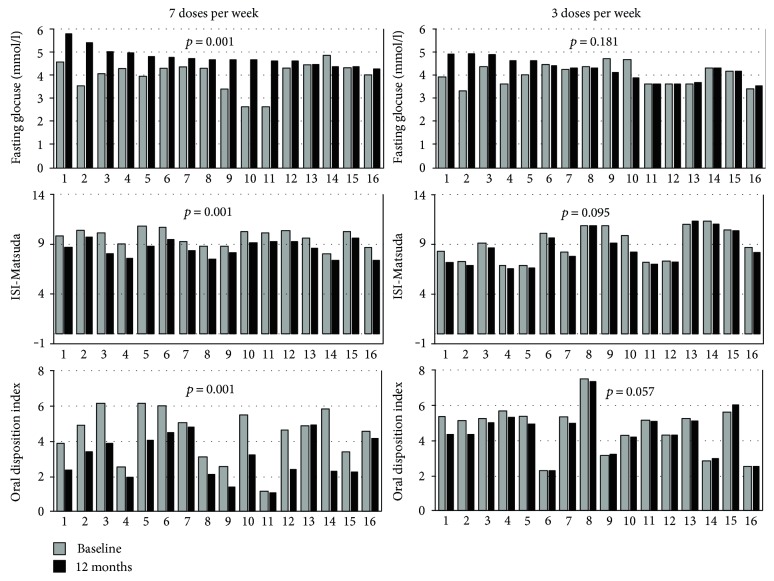
Fasting glucose, ISI-Matsuda, and oral disposition index (DIo) of patients grouped according to the GH treatment regimen (7 or 3 doses per week) at baseline and after 12 months of treatment.

As regards the metabolic parameters, no difference was found in baseline parameters between the 2 groups (Table 2). After 12 months of treatment in both groups, we found a significant increase in fasting insulin (9.2 ± 5.4 versus 2.9 ± 2.2 IU/mL; *p* < 0.001 and 5.7 ± 2.4 versus 2.8 ± 2.2 IU/mL; *p* = 0.026) and Homa-IR (1.98 ± 1.20 versus 0.52 ± 0.41 IU/mL; *p* < 0.001 and 1.16 ± 0.53 versus 0.53 ± 0.41; *p* = 0.019). A significant increase in fasting glucose (4.77 ± 0.39 versus 4 ± 0.65 mmol/L; *p* = 0.001) with a concomitant decrease in ISI-Matsuda (8.59 ± 0.83 versus 9.65 ± 0.82; *p* < 0.001) and DIo (2.46 ± 1.27 versus 4.48 ± 1.81; *p* = 0.002) from baseline to 12 months was only found in group A (Figure 1; Table 3).

No significant changes were found in either group in HbA1c levels and lipid profile (Table 3).

When we analyzed the change (delta) in clinical and metabolic parameters from baseline to 12 months of GHT, we found a significantly greater delta of insulin (*p* = 0.043), Homa-IR (*p* = 0.032), ISI-Matsuda (*p* < 0.001), and DIo (*p* = 0.001) in group A than in group B. The delta in fasting glucose and HbA1c, although not statistically significant, was found to be higher in group A than group B, while no significant difference was found in clinical parameters and in other metabolic indexes (Table 4). No side effects were observed in any child.

## 4. Discussion

This prospective and controlled study demonstrates that TIW regimen of GHT has comparable auxological effects to daily treatment with a more favorable metabolic impact, leading to a less pronounced worsening in insulin sensitivity and beta-cell secretion.

It is known that the classical GHT, consisting of a single daily GH injection, does not correspond to physiological spontaneous GH secretion and probably leads to continuous exposure and supraphysiological 24-hour integrated concentrations of GH. In addition, GH pulsatility reveals two major attributes, pulse amplitude and pulse frequency, and several studies have demonstrated that auxological parameters in children are mainly modulated by GH pulse amplitude, while frequency appears to play a smaller role [24, 25]. Clinical studies that have compared the TIW regimen with the daily administration of GH in children have always evaluated almost exclusively the auxological data, although the goal of GHT is also metabolic. Several studies report that the daily GH administration was more effective on linear growth than TIW dosing [2, 14, 16, 17, 26]. These data were confirmed by MacGillivray et al., who demonstrated greater growth velocity in prepubertal naïve GHD children after 4 years of GHT given daily rather than thrice weekly [27]. In our study, in line with previous studies, we found comparable auxological effects with the two GHT regimens, since all children showed a significant increase in height and growth velocity, without any difference in the delta of these parameters from baseline to 12 months of GHT in the two groups of children. These results are in agreement with the study of Smith et al., who demonstrated no difference in auxological parameters after 12 months of GHT in GHD children receiving GH three or six days a week or twice daily six days a week, assessing the main role played by the GH dose and not by the regimen of administration [14]. Similar results were shown a few years later by Cavallo et al., who observed no difference in auxological parameters during the 3 times/week treatment schedule started during the follow-up in already treated GHD children compared to the previous 6 times/week schedule [15]. The comparable auxological effect of the 2 regimens of GHT has also been demonstrated in patients with Turner syndrome randomized to 3 or 6 times weekly GHT [28]. In addition to auxological parameters, in our study after 12 months of GHT, IGF-I levels were shown to be similarly normalized in both groups, in line with other studies [29, 30], and they reinforce the idea of the noninferior clinical efficacy of TIW treatment compared to daily treatment.

Conversely, the metabolic effects of GHT given in an alternative regimen have been evaluated in adult patients and never in children. It is well known that severe GHD in children is associated with significant impairment of body composition and an adverse metabolic profile [31], as well as in adults [32].

Amato et al. showed that GHT with TIW regimen in adult GHD patients is effective in improving metabolic parameters, with an efficacy comparable to that observed in patients treated with daily GHT [29], and these data are in agreement with those of Giavoli et al. [33]. Reinforcing these findings, Pincelli et al. demonstrated that a TIW regimen of GHT is able to reverse cardiovascular abnormalities in addition to improving body composition and lipid profile in adult patients [34].

Substantially, our data demonstrated that all children, regardless of GHT regimen, showed a worsening in insulin sensitivity after 12 months of treatment, as demonstrated by the increase in Homa-IR, already shown in adult patients [33]. This effect of the GHT is well known. Indeed, given the insulin antagonistic action of GH in addition to the direct stimulatory effect on *β*-cell, GHT can lead to glucose metabolism impairment through a decrease in insulin sensitivity and an impairment of pancreatic *β*-cell function [35]. We previously demonstrated an increase in Homa-IR, related to the increased insulin levels, in GHD children after GHT [36, 37], and a degree of impairment of glucose metabolism during GHT has often been previously demonstrated [38]. However, the limit of the evaluation of the insulin sensitivity and secretion performed through insulin-derived indexes [39] is always to be taken into account.

As regards the change in glucose levels during GHT, in adult GHD patients, Johansson et al. demonstrated lower blood glucose and insulin levels during a TIW regimen than during daily injections [40]. The authors explained this result as due to the concomitant lower serum IGF-I levels, which may be important for the balanced effect of GH on insulin sensitivity and glucose metabolism [41].

Our data partially support this study. We only found a significant increase in fasting glucose levels in group A, despite the concomitant increase in insulin during GHT in both groups. This result suggests the inability of the *β*-cells to adequately compensate the insulin resistance state, as indicated by the significant reduction in DIo and ISI-Matsuda, only in group A. This finding is in agreement with a previous study that demonstrated inadequate *β*-cell compensation of decreased insulin sensitivity in GHD children during GHT [42].

In addition, confirming these data, when the change in metabolic parameters from baseline to 12 months of GHT was evaluated and directly compared in the 2 groups of children, group A showed a significantly greater decrease in insulin sensitivity and *β*-cell function than group B, with a concomitant greater increase in fasting glucose, although not statistically significant.

Probably, GHT using daily injections may not be optimal with respect to glucose metabolism because of the relative continuous exposure of GH. Furthermore, constant GH exposure might theoretically downregulate the GH receptor. Conversely, GHD children treated with the TIW regimen, because of the relatively short half-life of GH, have lower circulating GH during the “off” days and the more distinct peaks of GH could lead to a more favorable metabolic impact. Indeed, the lower metabolic effects should be advantageous for insulin sensitivity and, consequently, for fasting glucose levels, which did not significantly increase. However, the stable Hba1c levels indicate that there was no difference in long-term glucose homeostasis between the two regimens of GHT. This finding is in agreement with the data of Johansson et al., who showed comparable Hba1c levels in GHD adults after 8 weeks of GHT with TIW or daily regimen [40].

In addition, the current study shows no significant difference in lipid profile between the 2 groups of patients, in agreement with the study of Lucidi et al., who previously demonstrated that GHT given TIW-induced increments in protein synthesis and lipolysis comparable to those obtained after daily treatment GHT in adult patients [30].

On the other hand, GHT given as a TIW regimen could theoretically result in better patient compliance than daily injections and this point is relevant considering the need for long-term treatment [40]. However, we could not evaluate this issue because of the lack of the use of specific questionnaires for the assessment of quality of life or adherence to therapy, which is a limitation of our study. Other limitations of this study may be related to the small size of the population and the short-term follow-up. Indeed, although the best auxological benefits generally occur during the first year of treatment, a longer follow-up may be useful to have more reliable data. In addition, data on body composition measured by dual-energy X-ray absorptiometry as a marker of metabolic risk might be useful, but they are available for a minority of patients.

However, the discrepancies among the various studies are due to the high variability of the patients enrolled (i.e., the majority of the studies have enrolled children not naïve to GHT but already treated with GH for a different length of time), to different doses of GH used or different duration of the follow-up. To avoid these limits, our study was designed to enroll only GHD children naïve to GHT and consecutively admitted in a controlled way. To better understand whether the two regimens of GHT lead to different metabolic outcomes, we believe that these data must be validated in additional larger prospective studies with longer follow-up. Indeed, the duration of 12 months may be probably too short to fully define the metabolic impact of the two different modalities of treatment and these data should be considered as preliminary data.

In conclusion, our study confirmed that GHT with the TIW injections regimen is effective in improving auxological parameters and that the auxological efficacy of this regimen is comparable with that of a daily regimen. This regimen proved to have at least similar auxological effects to daily treatment, and it could theoretically ensure higher acceptance of treatment in children, in addition to having demonstrated for the first time a more favorable metabolic impact in GHD children. Therefore, the TIW injection regimen could represent an effective alternative to the conventional daily regimen during the first 1 year of GHT and can be favorably applied in the treatment of GHD children.

## Figures and Tables

**Table 1:** Clinical and biochemical features of patients grouped according to the GH treatment regimen (group A and group B) at baseline and after 12 months of treatment.

	7 doses per week (group A) 16 patients baseline	7 doses per week (group A) 16 patients 12 months		3 doses per week (group B) 16 patients baseline	3 doses per week (group B) 16 patients 12 months	
	Mean ± SD	Mean ± SD	*p*	Mean ± SD	Mean ± SD	*p*
Height (SD)	−2.06 ± 0.43	−1.5 ± 0.40	<0.001	−2.04 ± 0.69	−1.5 ± 0.5	<0.001
BMI (SD)	−0.46 ± 0.48	−0.45 ± 1.3	0.876	−0.79 ± 0.45	−0.86 ± 0.8	0.052
Waist circumference (cm)	60.5 ± 10.4	62 ± 10	0.317	60.1 ± 9.0	61.5 ± 7.0	0.463
Growth velocity (SD)	−3.4 ± 0.9	2.7 ± 1.7	0.003	−3.3 ± 0.9	3 ± 1.4	<0.001
Bone/chronological age ratio	0.81 ± 0.12	0.90 ± 0.05	0.089	0.81 ± 0.09	0.89 ± 0.11	0.098
IGF-I (*μ*g/L)	99.5 ± 23.8	332 ± 125	<0.001	100 ± 23.4	352 ± 89.1	<0.001

SD: standard deviation; BMI: body mass index.

**Table 2:** Difference in metabolic parameters between patients grouped according to the GH treatment regimen (group A and group B) at baseline.

	7 doses per week (group A) 16 patients	3 doses per week (group B) 16 patients	
	Mean ± SD	Mean ± SD	*p*
Fasting glucose (mmol/L)	4 ± 0.65	4.17 ± 0.45	0.801
Fasting insulin (IU/mL)	2.9 ± 2.2	2.8 ± 2.2	0.687
HbA1c (%)	5.1 ± 0.3	5.3 ± 0.3	0.108
Homa-IR	0.52 ± 0.41	0.53 ± 0.41	0.688
ISI-Matsuda	9.65 ± 0.82	9.03 ± 1.63	0.186
DIo	4.48 ± 1.81	4.68 ± 1.39	0.548
Total cholesterol (mmol/L)	4.12 ± 0.76	3.98 ± 0.51	0.130
HDL cholesterol (mmol/L)	1.58 ± 0.44	1.65 ± 0.31	0.563
LDL cholesterol (mmol/L)	2.84 ± 0.53	2.11 ± 0.36	0.063
Triglycerides (mmol/L)	1.50 ± 0.41	1.38 ± 0.52	0.942

BMI: body mass index; DIo: oral disposition index.

**Table 3:** Metabolic parameters of patients grouped according to the GH treatment regimen (group A and group B) at baseline and after 12 months of treatment.

	7 doses per week (group A) 16 patients baseline	7 doses per week (group A) 16 patients 12 months		3 doses per week (group B) 16 patients baseline	3 doses per week (group B) 16 patients 12 months	
	Mean ± SD	Mean ± SD	*p*	Mean ± SD	Mean ± SD	*p*
Fasting glucose (mmol/L)	4 ± 0.65	4.77 ± 0.39	0.001	4.17 ± 0.45	4.51 ± 0.35	0.181
Fasting insulin (IU/mL)	2.9 ± 2.2	9.2 ± 5.4	<0.001	2.8 ± 2.2	5.7 ± 2.4	0.026
HbA1c (%)	5.1 ± 0.3	5.2 ± 0.3	0.072	5.3 ± 0.3	5.2 ± 0.2	0.189
Homa-IR	0.52 ± 0.41	1.98 ± 1.20	<0.001	0.53 ± 0.41	1.16 ± 0.53	0.019
ISI-Matsuda	9.65 ± 0.82	8.59 ± 0.83	<0.001	9.03 ± 1.63	8.81 ± 1.57	0.095
DIo	4.48 ± 1.81	2.46 ± 1.27	0.001	4.68 ± 1.39	4.49 ± 1.30	0.057
Total cholesterol (mmol/L)	4.12 ± 0.76	4.07 ± 0.78	0.521	3.98 ± 0.51	3.87 ± 0.58	0.679
HDL cholesterol (mmol/L)	1.58 ± 0.44	1.54 ± 0.44	0.489	1.65 ± 0.31	1.86 ± 0.45	0.094
LDL cholesterol (mmol/L)	2.84 ± 0.53	2.84 ± 0.55	0.961	2.11 ± 0.36	1.95 ± 0.61	0.298
Triglycerides (mmol/L)	1.50 ± 0.41	1.54 ± 0.61	0.760	1.38 ± 0.52	1.22 ± 0.30	0.206

DIo: oral disposition index.

**Table 4:** Change (delta) in clinical and metabolic parameters from baseline to 12 months of GH treatment of patients grouped according to the GH treatment regimen.

	7 doses per week (group A) 16 patients	3 doses per week (group B) 16 patients	
	Mean ± SD	Mean ± SD	*p*
Delta height (SD)	0.5 ± 0.3	0.5 ± 0.2	0.926
Delta BMI (SD)	0.03 ± 0.6	−0.4 ± 0.6	0.109
Delta waist circumference (cm)	1.4 ± 4.4	1.4 ± 4.8	0.223
Delta growth velocity (SD)	5.2 ± 1.7	6.3 ± 2.7	0.945
Delta IGF-I (*μ*g/L)	220.5 ± 112.7	204.8 ± 70.9	0.210
Delta fasting glucose (mmol/L)	0.78 ± 0.76	0.34 ± 0.32	0.175
Delta fasting insulin (IU/mL)	6.3 ± 4.2	2.9 ± 3.4	0.043
Delta HbA1c (%)	0.07 ± 0.1	−0.05 ± 0.1	0.078
Delta Homa-IR	1.46 ± 0.99	0.63 ± 0.70	0.032
Delta ISI-Matsuda	−1.06 ± 0.43	−0.21 ± 0.19	<0.001
Delta DIo	−1.31 ± 0.97	−0.37 ± 0.34	0.001
Delta total cholesterol (mmol/L)	0.06 ± 0.01	0.08 ± 0.05	0.941
Delta HDL cholesterol (mmol/L)	0.04 ± 0.03	−0.12 ± 0.04	0.209
Delta LDL cholesterol (mmol/L)	0.01 ± 0.01	0.15 ± 0.04	0.498
Delta triglycerides (mmol/L)	0.04 ± 0.03	0.13 ± 0.05	0.392

BMI: body mass index; DIo: oral disposition index.
